# A Retrospective Analysis of the Predictive Role of RDW, MPV, and MPV/PLT Values in 28-Day Mortality of Geriatric Sepsis Patients: Associations with APACHE II and SAPS II Scores

**DOI:** 10.3390/medicina61081318

**Published:** 2025-07-22

**Authors:** Adem Koçak, Senem Urfalı

**Affiliations:** Department of Anesthesiology and Reanimation, Tayfur Ata Sokmen Faculty of Medicine, Hatay Mustafa Kemal University, Hatay 31060, Türkiye; kocak.adem@hotmail.com

**Keywords:** geriatrics, sepsis, mortality, acute physiology and chronic health evaluation, predictive value of tests, simplified acute physiology score, mean platelet volume

## Abstract

*Background and Objectives*: Immunodeficiency associated with aging comorbidities increases the vulnerability of geriatric patients to sepsis. Early recognition and management of sepsis are essential in this population. This study evaluated the relationships between RDW, MPV, and MPV/PLT ratios and mortality in geriatric sepsis patients. *Materials and Methods*: This retrospective study was conducted between 2020 and 2024 in the Intensive Care Unit of the Department of Anesthesiology and Reanimation at a university hospital. Patients aged ≥ 65 years with a SOFA score of ≥2 were included. Demographic data (sex, age, height, weight, and BMI), hemogram parameters (RDW, MPV, and PLT), blood gas, and biochemical values were analyzed. Furthermore, their comorbidities; site of infection; ICU length of stay; vital signs; and SOFA, APACHE II, and SAPS II scores, recorded within the first 24 h following ICU admission, were evaluated. Statistical analysis was performed using the chi-square test, Student’s *t*-test, the Mann–Whitney U test, the Monte Carlo exact test, and ROC analysis. A *p*-value of <0.05 was considered statistically significant. *Results*: A total of 247 patients were included, with 46.2% (n = 114) classified as non-survivors during the 28-day follow-up period. Among them, 64.9% (n = 74) were male, with a mean age of 78.22 ± 8.53 years. Significant differences were also found in SOFA, APACHE-II, and SAPS-II scores between non-survivors and survivors (SOFA: 7.64 ± 3.16 vs. 6.78 ± 2.78, *p* = 0.023; APACHE-II: 21.31 ± 6.36 vs. 19.27 ± 5.88, *p* = 0.009; SAPS-II: 53.15 ± 16.04 vs. 46.93 ± 14.64, *p* = 0.002). On days 1, 3, and 5, the MPV/PLT ratio demonstrated a statistically significant predictive value for 28-day mortality. The optimal cut-off values were >0.03 on day 1 (AUC: 0.580, 95% CI: 0.516–0.642, sensitivity: 72.81%, specificity: 65.91%, *p* = 0.027), >0.04 on day 3 (AUC: 0.602, 95% CI: 0.538–0.663, sensitivity: 60.53%, specificity: 60.61%, *p* = 0.005), and >0.04 on day 5 (AUC: 0.618, 95% CI: 0.554–0.790, sensitivity: 66.14%, specificity: 62.88%, *p* = 0.001). *Conclusions*: The MPV and MPV/PLT ratios demonstrated statistically significant but limited predictive value for 28-day mortality in geriatric patients with sepsis. In contrast, the limited prognostic value of RDW may be related to variability in the inflammatory response and other underlying conditions. The correlations found between SOFA, APACHE II, and SAPS II scores highlight their importance in mortality risk prediction.

## 1. Introduction

Sepsis is defined as life-threatening organ dysfunction resulting from the body’s dysregulated response to infection [1]. According to data from 2020, sepsis was responsible for 48.9 million cases and 11 million deaths globally, corresponding to 20% of all deaths [2]. Mortality rates for severe sepsis remain high, with approximately 30% of patients not surviving [3]. The type and virulence of pathogens causing sepsis may vary depending on the source of infection; in many cases, gram-negative bacteria predominate. In addition, invasive procedures and medical devices increase the risk of developing infections with resistant organisms [4].

High-risk groups for sepsis include children, elderly patients, individuals with chronic illnesses (e.g., cancer or diabetes), and immunosuppressed individuals [4]. In the elderly, age-related physiological changes and chronic diseases significantly increase the risk of infection. In hospitalized patients aged 65 years and older, mortality rates range from 30% to 60%, while in patients aged 80 years and above, these rates can increase to 80% [5]. This underscores need for effective risk stratification and early prognostic markers in the geriatric population.

Several scoring systems, including the Acute Physiology and Chronic Health Evaluation (APACHE) II, III, and IV; the Simplified Acute Physiology Score (SAPS); the Sequential Organ Failure Assessment (SOFA); and the Multiple Organ Dysfunction Score (MODS) have been developed to assess prognosis and determine the severity of organ dysfunction in patients with sepsis [6,7]. Among them, the APACHE II score is widely used to evaluate mortality risk in Intensive Care Unit (ICU) patients and is calculated within the first 24 h of ICU admission [8,9]. Similarly, SAPS II, also calculated within the first 24 h, was developed as an alternative to APACHE and provides an estimated mortality rate ranging from 0% to 100% [6]. The SOFA score is a crucial tool for diagnosing and predicting the progression of organ dysfunction in sepsis. It also emphasizes the importance of evaluating the relationship between chronic comorbidities and acute organ failure [1]. These scoring systems emphasize the importance of rapid classification; however, they often rely on multiple clinical and laboratory parameters, which may limit their applicability to frail elderly patients. This has led researchers to investigate simpler blood-based biomarkers that are readily available at the time of admission for additional prognostic information.

Red cell distribution width (RDW) evaluates the variability in red blood cell size and has been associated with harmful pathological processes including inflammation, oxidative stress, and malnutrition during critical illness. The evaluation of RDW has been utilized as a valuable prognostic biomarker in septic patients [10,11]. Recent studies suggest that RDW is significantly associated with sepsis-related mortality. Moreover, its prognostic performance in predicting mortality has been found to be comparable to established scoring systems such as the Sepsis-related Organ Failure Assessment (SOFA) and the Acute Physiology and Chronic Health Evaluation II (APACHE II) [12,13]. However, the literature is not uniform on this point, and the role of RDW remains debated. According to the findings of Zhang et al. and Laukemann et al., RDW may not serve as a dependable biomarker for predicting outcomes in sepsis [14,15,16]. While RDW has been increasingly investigated as an independent prognostic marker, inconsistent findings across studies have limited its adoption in clinical settings. Therefore, its prognostic role remains uncertain and requires further validation.

Mean platelet volume (MPV) reflects platelet activation and aggregation, and it has been associated with prognosis in inflammatory conditions such as sepsis. Recent studies have suggested that it may serve as a useful indicator of sepsis severity and mortality [17,18]. Mean platelet volume is an appealing marker because it is automatically reported as part of the complete blood count and thus available at no extra cost or time. However, its reliability as an inflammatory marker and its relationship with disease severity remain controversial [19,20]. Some studies indicate that MPV increases during severe sepsis, whereas others have observed decreases or no clear pattern [16,21,22]. In other words, while certain cohorts show a trend of elevated MPV correlating with worse prognosis, other studies did not find MPV alone to be a dependable predictor of sepsis outcome. At present, there is no consensus on MPV’s prognostic value due to these mixed results, and it remains a subject of debate and active investigation.

Platelets (PLT) play a key role in inflammation by facilitating leukocyte adhesion and extravasation while maintaining vascular stability. They also regulate neutrophil and monocyte functions, increasing the inflammatory response. Through these mechanisms, platelets contribute to excessive inflammation and the progression of sepsis by mediating both inflammatory and thrombotic pathways [23]. Thrombocytopenia is common in septic patients and is incorporated into the SOFA score as an indicator of organ dysfunction [1,16,24]. A failure of platelet counts to recover during sepsis is associated with higher mortality, whereas platelet count recovery correlates with better outcomes [1,16]. In this context, indices derived from platelets (like MPV or platelet distribution width) are biologically plausible markers of disease severity, but as noted above, their prognostic performance is still being clarified.

This study hypothesized that RDW, MPV, and MPV/PLT ratios are significant prognostic markers for predicting 28-day mortality in geriatric sepsis patients. Hence, this study was designed to answer this research question to evaluate the prognostic value of these parameters and investigate their associations with APACHE II and SAPS II scores to understand their potential impact on sepsis outcomes.

## 2. Materials and Methods

### 2.1. Study Design

This study was designed as a retrospective analysis of patients admitted to the Intensive Care Unit of the Department of Anesthesiology and Reanimation at the Faculty of Medicine, Hatay Mustafa Kemal University. The study was approved by the Non-Interventional Research Ethics Committee of Hatay Mustafa Kemal University (Approval No: 05, dated 4 March 2024) and conducted in accordance with the ethical principles of the Declaration of Helsinki. Due to its retrospective design, informed consent was not obtained, and all data were anonymized prior to analysis.

The medical records of patients treated between 1 January 2020 and 1 January 2024 were reviewed. All patient data were retrieved from the Hospital Information Management System (HIMS) and were cross-checked with original patient files by two independent researchers to ensure accuracy and completeness. Cases with missing or inconsistent data were excluded during the initial screening process.

The study included geriatric patients aged 65 years and older with a Sequential Organ Failure Assessment (SOFA) score of 2 or higher. Exclusion criteria included patients under the age of 65 and those with a SOFA score below 2; missing data; or a diagnosis of chronic hepatitis or cirrhosis, hematologic malignancies, or immunosuppressive conditions. Demographic data (sex, age, height, weight, and BMI); hemogram parameters (RDW, MPV, and PLT); arterial blood gas; and biochemical values including pH, HCO_3_, lactate, BUN, creatinine, and CRP, obtained at the time of ICU admission, were analyzed. Additionally, their comorbidities; site of infection; ICU length of stay; vital signs; and SOFA, APACHE II, and SAPS II scores, recorded within the first 24 h of ICU admission, were evaluated. The RDW, MPV, and MPV/PLT ratios, obtained from complete blood count tests, were retrospectively evaluated based on laboratory records from the day of ICU admission and on the 3rd and 5th days of ICU stay. Changes in these values and their association with 28-day mortality were analyzed.

**Figure 1:**
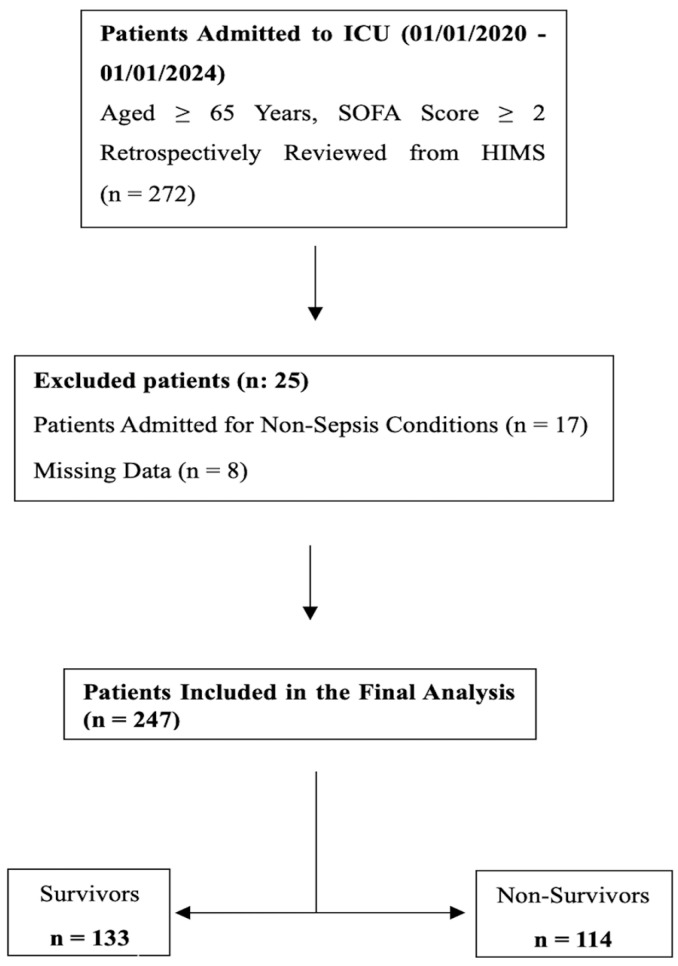
Study Flowchart.

In accordance with the cross-sectional study design, a total of 247 patients who were admitted to the Intensive Care Unit of the Department of Anesthesiology and Reanimation between January 2020 and January 2024, with a SOFA score ≥ 2 and aged ≥ 65 years, were included in the study. A total of 272 patients were initially screened based on the inclusion criteria. After excluding patients with non-sepsis-related ICU admissions (n = 17) and those with insufficient data (n = 8), 247 patients were included in the final analysis. These patients were then classified into two groups based on their 28-day outcomes: survivors (n = 133) and non-survivors (n = 114). A flowchart detailing the process of patient selection and classification is presented in Figure 1.

### 2.2. Statistical Analysis

The collected data were recorded in a digital database and analyzed using the IBM SPSS V.22 software. Categorical data were presented as frequencies and percentages, while numerical data were described based on their distribution characteristics.

Numerical data following a normal distribution were expressed as mean ± standard deviation (SD), whereas data not conforming to a normal distribution were presented as median and interquartile range (IQR). Categorical data were compared using the chi-square test and Monte Carlo exact test. For numerical data, the Student’s *t*-test was used for normally distributed variables, and the Mann–Whitney U test was used for non-normally distributed variables.

Receiver operating characteristic (ROC) analysis was performed to evaluate the predictive power for survival. A *p*-value of <0.05 was considered statistically significant in all analyses.

## 3. Results

A total of 247 patients aged 65 years and older were included in the study, with a mean age of 77.3 ± 8.8 years. The demographic and clinical characteristics of survivors and non-survivors are presented in Table 1.

No statistically significant differences were observed between survivors and non-survivors in terms of age (76.57 ± 9.02 vs. 78.22 ± 8.53; t:1.475; *p* = 0.141), height (165.96 ± 8.07 cm vs. 167.84 ± 9.05 cm; t: 1.718; *p* = 0.087), weight (72.07 ± 13.44 kg vs. 72.96 ± 12.83 kg; t: 0.529; *p* = 0.597), or BMI (26.05 ± 3.79 vs. 25.84 ± 3.81; t: 0.433; *p* = 0.666).

Pneumosepsis was the most common source of infection, observed in 53.4% (n = 71) of survivors and 61.4% (n = 70) of non-survivors ($X^{2}$: 3.196; *p* = 0.110). Gastrointestinal infections were found in 18.8% (n = 25) of survivors and 10.5% (n = 12) of non-survivors, while other infection sites (urosepsis, catheter-related sepsis, or skin-and-soft-tissue-related sepsis) were reported at similar rates between the groups (27.8% vs. 28.1%).

A significant difference in gender distribution was observed between the groups. In the survivor group, 47.7% (n = 63) were female and 52.6% (n = 70) were male. In contrast, in the non-survivor group, 35.1% (n = 40) were female and 64.9% (n = 74) were male ($X^{2}$: 3.793; *p* = 0.015). ICU length of stay was also significantly different, with survivors staying for 22.49 ± 8.70 days compared to 16.74 ± 7.02 days for non-survivors (t: 5.650; *p* < 0.001). The Glasgow Coma Scale (GCS) score was significantly higher in survivors (8.67 ± 2.91) compared to non-survivors (7.78 ± 3.04) (t: 0.902; *p* = 0.019).

The distribution of chronic diseases is presented in Table 2. Significant differences were observed between survivors (n = 133) and non-survivors (n = 114) regarding hypertension and diabetes mellitus. The prevalence of diabetes mellitus was significantly higher in survivors at 42.1% (n = 56) compared to 28.9% (n = 33) in non-survivors ($X^{2}$: 4.592; *p* = 0.011). In contrast, hypertension was more common in non-survivors (34.2%, n = 39) than in survivors (24.1%, n = 32) ($X^{2}$: 3.075; *p* = 0.024). No significant differences were found between the groups regarding the prevalence of coronary artery disease (36.1%, n = 42 vs. 34.2%, n = 39; $X^{2}$: 0.095; *p* = 0.101), chronic kidney failure (15.8%, n = 21 vs. 21.9%, n = 25; $X^{2}$: 1.571; *p* = 0.061), or COPD (9.8%, n = 13 vs. 14.9%, n = 17; $X^{2}$: 1.512; *p* = 0.073). Similarly, cerebrovascular disease and neoplasm showed no statistically significant differences between survivors (22.6%, n = 30 and 21.1%, n = 28) and non-survivors (17.5%, n = 20 and 16.7%, n = 19) ($X^{2}$: 0.951; *p* = 0.079 and $X^{2}$: 0.865; *p* = 0.089, respectively).

No significant differences were observed between the groups regarding heart rate (*p* = 0.083), systolic blood pressure (SBP) (*p* = 0.213), diastolic blood pressure (DBP) (*p* = 0.786), or mean arterial pressure (MAP) (*p* = 0.434). Similarly, oxygen saturation (SpO_2_), body temperature, and respiratory rate did not differ significantly between survivors and non-survivors (*p* = 0.933, *p* = 0.103, *p* = 0.201, respectively).

MPV values were significantly higher in non-survivors than in survivors across all measured days. On day 1, MPV was 10.22 ± 1.42 in survivors and 10.58 ± 1.43 in non-survivors (t: 1.994; *p* = 0.047). On day 3, these values were 10.20 ± 1.40 and 10.86 ± 1.49, respectively (t: 4.164; *p* < 0.001), while on day 5, MPV was 10.17 ± 1.27 in survivors compared to 10.93 ± 1.53 in non-survivors (t: 2.148; *p* = 0.011). No significant differences were found between the groups regarding WBC count, hemoglobin, hematocrit, RDW, or PLT values (*p* > 0.05) (Table 3).

Table 4 presents a comparison of MPV/PLT ratios and SOFA, APACHE-II, and SAPS-II scores between survivors and non-survivors. Significant differences were observed in MPV/PLT ratios across all measured days. On day 1, the ratio was 0.041 (IQR: 0.03) in survivors and 0.047 (IQR: 0.04) in non-survivors (U: 6359; *p* = 0.029). On day 3, it was 0.039 (IQR: 0.03) in survivors and 0.047 (IQR: 0.04) in non-survivors (U: 6084.5; *p* = 0.008), and on day 5, the ratio was 0.037 (IQR: 0.02) in survivors and 0.047 (IQR: 0.05) in non-survivors (U: 5832; *p* = 0.002).

Significant differences were also found in SOFA, APACHE-II, and SAPS-II scores. The SOFA score was 6.78 ± 2.78 in survivors and 7.64 ± 3.16 in non-survivors (t: 2.290; *p* = 0.023). The APACHE-II score was 19.27 ± 5.88 in survivors and 21.31 ± 6.36 in non-survivors (t: 2.622; *p* = 0.009), while the SAPS-II score was 46.93 ± 14.64 in survivors and 53.15 ± 16.04 in non-survivors (t: 3.186; *p* = 0.002).

Significant differences were observed between survivors and non-survivors regarding pH (7.40 ± 0.09 vs. 7.36 ± 0.12; t: 2.982; *p* = 0.002), HCO_3_ (24.88 ± 7.46 vs. 22.37 ± 7.19; t: 2.682; *p* = 0.008), lactate (2.66 ± 2.65 vs. 3.5 ± 3.09; t: 2.316; *p* = 0.021), BUN (40.39 ± 25.28 vs. 49.67 ± 30.62; t: 2.608; *p* = 0.010), creatinine (1.00 [1.04] vs. 1.42 [1.16]; U: 5771.5; *p* = 0.003), and CRP (106.67 ± 64.08 vs. 129.02 ± 71.31; t: 2.593; *p* = 0.010). In contrast, no statistically significant differences were found regarding PaCO_2_, FiO_2_, PaO_2_, Na, K, total bilirubin, or albumin levels (*p* > 0.05) (Table 5).

**Figure 2:**
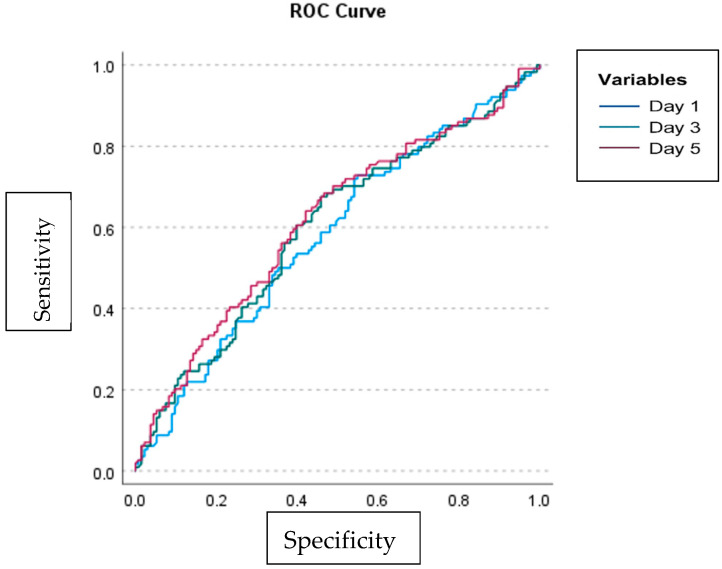
ROC curves for MPV/PLT values measured on the 1st, 3rd, and 5th days for predicting 28-day mortality.

To evaluate the performance of the MPV/PLT ratio in predicting 28-day mortality, ROC analysis was conducted, and the cut-off values, sensitivity, specificity, AUC, and *p*-values were determined for each measurement day. On the first day, with a cut-off value of >0.03, the sensitivity and specificity of the MPV/PLT ratio were found to be 72.81% and 65.91%, respectively; the AUC was 0.580 (95% CI: 0.516–0.642), with a *p*-value of 0.027. On the third day, using a cut-off of >0.04, sensitivity was 60.53%, specificity was 60.61%, AUC was 0.602 (95% CI: 0.538–0.663), and the *p*-value was 0.005. On the fifth day, again with a cut-off of >0.04, sensitivity was 66.14%, specificity was 62.88%, and the AUC was 0.618 (95% CI: 0.554–0.790), with a *p*-value of 0.001. The ROC curves are presented in Figure 2, and detailed cut-off characteristics are shown in Table 6.

## 4. Discussion

Geriatric patients represent a significant part of the population and are particularly vulnerable to various diseases due to their frail physical condition. The present study aimed to evaluate the prognostic significance of RDW, MPV, and MPV/PLT ratios in predicting 28-day mortality among geriatric sepsis patients and their associations with SOFA, APACHE II, and SAPS II scores. The findings revealed that MPV and MPV/PLT ratios were significantly associated with increased mortality, whereas RDW demonstrated limited prognostic value.

In a study involving 163 patients, Vélez-Páez et al. found that MPV and the MPV/PLT ratio were significant predictors of sepsis severity and mortality [17]. Similarly, Ho Oh et al. analyzed early mortality in 120 patients with severe sepsis and reported that MPV and PLT values alone were not effective for predicting shock, but the MPV/PLT ratio on the first day was a strong predictor of 28-day mortality [25]. A study by Ho Kim et al. highlighted that elevated MPV levels in patients with sepsis and septic shock were an important risk factor during hospital follow-up and were predictive of mortality [26]. Djordjevic et al. reported that MPV/PLT ratios were significantly higher (*p* < 0.01) in non-survivors with sepsis and/or trauma in the surgical ICU [21,27].

In our study, elevated mean MPV values were observed in non-survivors across all three days (*p*-values: 0.047, <0.001, and 0.011 for days 1, 3, and 5, respectively), while no significant differences were observed in PLT values between the groups. Our findings are consistent with previous studies reporting elevated MPV as a risk factor for mortality in sepsis [26]. In addition, the cut-off values of the MPV/PLT ratios on the first, third, and fifth days were found to be significant in predicting non-survivors (*p* values: 0.027, 0.005, and 0.001, respectively). Although the MPV/PLT ratio demonstrated statistically significant discriminatory power with defined cut-off values for all three days, the relatively low AUC (area under the curve) values indicate limited clinical utility. These findings support the clinical feasibility of integrating MPV- and PLT-derived indices into ICU protocols. Their cost-effectiveness and rapid availability at no extra expense make them attractive tools for initial sepsis screening, especially in geriatric ICU settings.

Various predictive scoring systems are used to estimate mortality. In a study conducted by Morkar et al. involving 100 patients aged 60–79, the relationships between SOFA, APACHE, and SAPS scores and the mortality of sepsis patients were examined, and these scores were found to predict mortality with high sensitivity [28]. Similarly, in a study by Tekin et al. including 202 patients, it was reported that SOFA, APACHE II, and SAPS II scores were successful in predicting 28-day mortality [29]. In a recent cohort study by Hu et al., SAPS II was shown to have the highest predictive accuracy for 30-day mortality in ICU patients with sepsis when compared to other widely used scoring systems such as LODS, SIRS, and SOFA. This finding supports its continued clinical relevance, particularly in geriatric populations [30]. In our study, SOFA, APACHE II, and SAPS II scores were also used to predict 28-day mortality, and these scores were significantly higher in non-survivors. These findings, consistent with studies in the literature, demonstrate that these scores are reliable parameters for predicting mortality and should be regularly monitored in patient management.

A study by Kim et al. involving 458 geriatric patients examined the correlation between RDW and 30-day mortality and highlighted RDW as an independent risk factor [31]. Similarly, Wang et al., in a study with 117 patients, found that RDW was an independent predictor of in-hospital mortality [32]. Park et al. demonstrated that RDW levels were significantly elevated in septic patients compared to healthy individuals. Moreover, RDW showed strong diagnostic performance in identifying sepsis [33]. However, in our study, no significant differences in RDW values were observed between survivors and non-survivors. This difference is likely due to the limited sample size in our study, despite RDW being considered an independent risk factor in the literature. Additionally, unmeasured confounders such as patients’ nutritional status, underlying types of anemia, or deficiencies in iron, folate, or vitamin B12 may have influenced RDW variability in our study, which could not be systematically evaluated due to the retrospective design.

It is known that conditions such as diabetes mellitus (DM), heart failure, and the need for mechanical ventilation increase mortality [34]. In a study by Ahlberg et al., hypertension (HT), DM, and socioeconomic factors were highlighted as being associated with sepsis and mortality [35]. In this study, analyses of chronic diseases revealed that HT was more frequent in the non-survivor group compared to the survivor group while DM was less common. These findings are thought to be related to the immunological changes caused by underlying pathologies.

Bicarbonate plays a critical role in the management of sepsis and septic shock. In the 2023 update, it was emphasized that bicarbonate levels should be evaluated and supported on a patient-specific basis [36]. In a study involving 424 patients with sepsis, impaired capillary refill, loss of peripheral circulation, and elevated lactate levels were shown to be associated with increased mortality [37]. In our study, blood gas analysis revealed that lactate levels were significantly higher and bicarbonate levels were significantly lower in non-survivors. This finding is consistent with the data in the literature that describe peripheral circulation impairment and bicarbonate deficit in advanced sepsis.

Biomarkers are essential in the diagnosis and management of sepsis, as they help reveal underlying pathophysiological processes and support patient classification for personalized treatment. Elevated levels of biomarkers such as serum amyloid A (SAA), sTREM-1, mannan, anti-mannan antibodies, interleukin-6 (IL-6), interleukin-8 (IL-8), MCP-1, presepsin, and suPAR have been linked to sepsis and are commonly used for early diagnosis and prognostic evaluation [38,39]. Conventional markers like white blood cell count (WBC), C-reactive protein (CRP), and procalcitonin (PCT) have long been used in clinical practice to identify sepsis [40,41]. A recent large-scale multicenter randomized clinical trial compared procalcitonin (PCT)- and C-reactive protein (CRP)-guided monitoring protocols in critically ill patients with suspected sepsis. The study demonstrated that daily PCT-guided protocols significantly reduced antibiotic duration compared to standard care [42].

Our study has several limitations. The primary limitation is its single-center, retrospective design, which may limit the generalizability and external validity of the findings. Additionally, the inability to fully control the data collection process and the relatively small sample size may have affected the accuracy of the results. Future prospective multicenter studies with larger and more diverse patient populations are needed to validate and broaden the applicability of our findings. Lastly, the exclusion of patients with certain underlying conditions, such as hematologic malignancies or chronic liver diseases, may have limited the relevance of the results to a broader patient population.

## 5. Conclusions

This study highlights the significance of clinical and laboratory parameters such as the MPV/PLT ratio, lactate, and bicarbonate levels in predicting mortality among geriatric sepsis patients. The significantly higher MPV/PLT ratio observed in non-survivors suggests its potential as a valuable prognostic marker. Additionally, MPV and MPV/PLT values obtained from routine complete blood counts, along with APACHE II and SAPS II scores, are identified as low-cost and effective predictors.

However, prospective multicenter studies with larger patient groups are needed to confirm their predictive value and assess their clinical use. Moreover, the integration of MPV and PLT measurements into clinical risk scoring systems, their incorporation into electronic health record platforms, and cost-effectiveness evaluations should be explored to determine their applicability in ICU practice.

## Figures and Tables

**Table 1:** Demographic and clinical characteristics of survivors and non-survivors.

Variable	Survivors (n = 133)	Non-Survivors (n = 114)	Test Statistic Value	*p*-Value
**Gender (n, %)**				
Female	63 (47.7)	40 (35.1)	$X^{2}$**:** **3.793**	**0.015**
Male	70 (52.6)	74 (64.9)		
Age (years, mean ± SD)	76.57 ± 9.02	78.22 ± 8.53	t: 1.475	0.141
Height (cm, mean ± SD)	165.96 ± 8.07	167.84 ± 9.05	t: 1.718	0.087
Weight (kg, mean ± SD)	72.07 ± 13.44	72.96 ± 12.83	t: 0.529	0.597
BMI (kg/m^2^, mean ± SD)	26.05 ± 3.79	25.84 ± 3.81	t: 0.433	0.666
**Site of infection, n (%)**				
Pneumosepsis	71 (53.4%)	70 (61.4%)	$X^{2}$: 3.196	0.110
Gastrointestinal tract	25 (18.8%)	12 (10.5%)		-
Other	37 (27.8%)	32 (28.1%)		-
ICU length of stay (days, mean ± SD)	22.49 ± 8.70	16.74 ± 7.02	**t: 5.650**	**<0.001**
GCS	8.67 ± 2.91	7.78 ± 3.04	**t: 0.902**	**0.019**

$X^{2}$: chi-square test, t: Student’s *t* test, BMI: Body Mass Index, ICU: Intensive Care Unit, GCS: Glasgow Coma Scale.

**Table 2:** The distribution of chronic diseases in survivors (n = 133) and non-survivors (n = 114).

Chronic Disease	Survivors (n, %)	Non-Survivors (n, %)	Test Statistic Value	*p*-Value
Diabetes mellitus	56 (42.1)	33 (28.9)	$X^{2}$ **: 4.592**	**0.011**
Hypertension	32 (24.1)	39 (34.2)	$X^{2}$ **: 3.075**	**0.024**
Coronary artery disease	42 (36.1)	39 (34.2)	$X^{2}$ **: 0.095**	0.101
Chronic kidney failure	21 (15.8)	25 (21.9)	$X^{2}$ **: 1.571**	0.061
COPD	13 (9.8)	17 (14.9)	$X^{2}$ **: 1.512**	0.073
Cerebrovascular disease	30 (22.6)	20 (17.5)	$X^{2}$ **: 0.951**	0.079
Neoplasm	28 (21.1)	19 (16.7)	$X^{2}$ **: 0.865**	0.089

$X^{2}$: chi-square test, COPD: Chronic Obstructive Pulmonary Disease.

**Table 3:** Comparison of blood count parameters by day and survival Status.

	Variable	Survivors (n = 133)	Non-Survivors (n = 114)	Test Statistic Value	*p*-Value
**WBC (10^3^/mm^3^)**	Day 1 (Median, IQR)	12.86 (9.36)	12.30 (6.30)	U: 7569.5	0.984
	Day 3 (Mean ± SD)	13.30 ± 5.71	13.97 ± 6.24	t: 0.146	0.884
	Day 5 (Mean ± SD)	14.23 ± 9.06	15.05 ± 6.58	t: 0.804	0.422
**Hemoglobin (g/d** **L** **)**	Day 1 (Mean ± SD)	10.66 ± 2.31	10.24 ± 2.44	t: 0.775	0.439
	Day 3 (Mean ± SD)	10.24 ± 2.03	10.49 ± 2.33	t: 0.890	0.374
	Day 5 (Mean ± SD)	10.14 ± 1.96	10.35 ± 2.08	t: 0.813	0.417
**Hematocrit (%)**	Day 1 (Mean ± SD)	32.79 ± 6.92	33.63 ± 7.08	t: 0.940	0.345
	Day 3 (Mean ± SD)	31.72 ± 5.73	32.45 ± 5.73	t: 0.918	0.360
	Day 5 (Mean ± SD)	31.35 ± 5.56	32.22 ± 6.08	t: 1.164	0.245
**RDW**	Day 1 (Mean ± SD)	16.17 ± 2.84	16.96 ± 3.08	t: 0.543	0.588
	Day 3 (Mean ± SD)	16.10 ± 2.91	15.80 ± 2.78	t: 0.816	0.415
	Day 5 (Mean ± SD)	16.23 ± 2.85	16.21 ± 2.75	t: 0.098	0.955
**MPV**	Day 1 (Mean ± SD)	10.22 ± 1.42	10.58 ± 1.43	t: 1.994	**0.047**
	Day 3 (Mean ± SD)	10.20 ± 1.40	10.86 ± 1.49	t: 4.164	**<0.001**
	Day 5 (Mean ± SD)	10.17 ± 1.27	10.93 ± 1.53	t: 2.148	**0.011**
**PLT**	Day 1 (Mean ± SD)	270.66 ± 148.11	242.41 ± 147.18	t: 1.499	0.135
	Day 3 (Mean ± SD)	271.98 ± 152.49	243.80 ± 155.01	t: 1.437	0.152
	Day 5 (Mean ± SD)	271.13 ± 135.62	238.19 ± 152.50	t: 1.742	0.086

U: Mann–Whitney U test, t: Student’s *t* test, WBC: white blood cell, RDW: red cell distribution width, MPV: mean platelet volume, PLT: platelet.

**Table 4:** Comparison of MPV/PLT ratios and SOFA, APACHE-II, and SAPS-II scores between survivors and non-survivors.

Variable		Survivors (n = 133)	Non-Survivors (n = 114)	Test Statistic Value	*p*-Value
MPV/PLT	Day 1 (Median, IQR)	0.041 (0.03)	0.047 (0.04)	U: 6359	**0.029**
	Day 3 (Median, IQR)	0.039 (0.03)	0.047 (0.04)	U: 6084.5	**0.008**
	Day 5 (Median, IQR)	0.037 (0.02)	0.047 (0.05)	U: 5832	**0.002**
SOFA Score (Mean ± SD)		6.78 ± 2.78	7.64 ± 3.16	**t: 2.290**	**0.023**
APACHE-II Score (Mean ± SD)		19.27 ± 5.88	21.31 ± 6.36	**t: 2.622**	**0.009**
SAPS-II Score (Mean ± SD)		46.93 ± 14.64	53.15 ± 16.04	**t: 3.186**	**0.002**

U: Mann–Whitney U test, t: Student’s *t* test, MPV: mean platelet volume, PLT: platelet, SOFA: Sequential Organ Failure Assessment, APACHE: Acute Physiology and Chronic Health Evaluation, SAPS: Simplified Acute Physiology Score.

**Table 5:** Comparison of blood gas and biochemical values based on survival status.

Variable	Survivors (Mean ± SD or Median (IQR))	Non-Survivors (Mean ± SD or Median (IQR))	Test Statistic Value	*p*-Value
**pH**	7.40 ± 0.09	7.36 ± 0.12	**t: 2.982**	**0.002**
**PaCO_2_ (mmHg)**	40.51 ± 13.75	39.10 ± 10.86	t: 0.887	0.376
**FiO_2_ (%)**	59.10 ± 19.04	59.49 ± 17.36	t: 0.165	0.869
**PaO_2_ (mmHg)**	109.46 ± 49.04	110.68 ± 44.54	t: 0.205	0.838
**HCO_3_ (mmol/L)**	24.88 ± 7.46	22.37 ± 7.19	**t: 2.682**	**0.008**
**Lactate (mmol/L)**	2.66 ± 2.65	3.50 ± 3.09	**t: 2.316**	**0.021**
**Na (mmol/L)**	139.49 ± 6.42	138.30 ± 7.38	t: 1.354	0.177
**K (mmol/L)**	4.18 ± 0.80	4.33 ± 0.92	t: 1.409	0.160
**Total Bilirubin (mg/dL)**	0.80 (0.38)	0.80 (0.44)	U: 7517	0.909
**BUN (mg/dL)**	40.39 ± 25.28	49.67 ± 30.62	**t: 2.608**	**0.010**
**Creatinine (mg/dL)**	1 (1.04)	1.42 (1.16)	**U: 5771.5**	**0.003**
**CRP (mg/L)**	106.67 ± 64.08	129.02 ± 71.31	**t: 2.593**	**0.010**
**Albumin (g/dL)**	3 (0.80)	2.90 (0.63)	U: 7123	0.411

U: Mann–Whitney U test, t: Student’s *t* test.

**Table 6:** Determination of cut-off value at MPV/PLT values measured on days 1, 3, and 5 for 28-day mortality prediction.

MPV/PLT	AUC (%95 CI: Lower–Upper)	Cut-Off	Sensitivity (%)	Specificity (%)	*p*-Value
**Day 1**	0.580 (0.516–0.642)	>0.03	72.81	65.91	**0.027**
**Day 3**	0.602 (0.538–0.663)	>0.04	60.53	60.61	**0.005**
**Day 5**	0.618 (0.554–0.79)	>0.04	66.14	62.88	**0.001**

AUC: Area under the curve, CI: Confidence interval.

## Data Availability

The datasets generated during and/or analyzed during the current study are available from the corresponding author upon reasonable request.

## Abbreviations

The following abbreviations are used in this manuscript:

MRSA	Methicillin-resistant staphylococcus aureus
APACHE	Acute Physiology and Chronic Health Evaluation
SAPS	Simplified Acute Physiology Score
SOFA	Sequential Organ Failure Assessment
MODS	Multiple Organ Dysfunction Score
ICU	Intensive Care Unit
RDW	Red cell distribution width
MPV	Mean platelet volume
PLT	Platelets
HIMS	Hospital Information Management System
GCS	Glasgow Coma Scale
SBP	Systolic blood pressure
DBP	Diastolic blood pressure
MAP	Mean arterial pressure
DM	Diabetes mellitus
HT	Hypertension
COPD	Chronic Obstructive Pulmonary Disease
WBC	White blood cell
BUN	Blood Urea Nitrogen
CRP	C-reactive protein
